# Modern "Bills of Mortality"

**Published:** 1923-12

**Authors:** 


### HOSPITAL and HEALTH REVIEW

### MODERN "BILLS OF MORTALITY."

We no longer talk, as our grandfathers did, of the "Bills of Mortality"; in language more precise and scientific we call them "Vital Statistics." The change of idiom is more significant than would appear at first sight, since it is rapidly becoming more in accord with the facts to speak of bills of life and health than of mortality. The Registrar-General's Statistical Review of England and Wales for 1922, the First Part of which has now been published well ahead of the usual date, is a document which, taken in conjunction with other official figures, fully justifies the remark. It is true that the death-rate, at 12.8 per thousand, was a fraction higher than that of 1921 when it was only 12.1; but the epidemic of influenza was responsible for nearly one-half of the increase. Against this we may set the very remarkable fact that the present year promises to be the healthiest on record, the death-rate for the first nine months having little exceeded ten per thousand. The further reduction of infant mortality, from 97 in 1918, 89 in 1919, 80 in 1920, and 83 in 1921 to 77 in 1922 has had an important influence upon the total death-rate. Fewer young children, in proportion to population, died last year than in any twelve months since we began to keep accurate statistics. Even in healthy France the death-rate in 1921 exceeded 17 per thousand, while the infant rate was 115. In Spain, in the same year, the total rate was 21, and the infant rate 147. Happily we do not stand alone in improvement, since as recently as 1918 Spain's total mortality rate was 33, and its infant rate 183. Yet we have hitherto looked upon that country as in its sanitary infancy.

We are entitled to congratulate ourselves upon the results of the steady hard work and ceaseless preaching of myriads of medical men and sanitarians, and the labours of innumerable organisations for enforcing the duty of men to be healthy themselves and to induce health in others; but no social reformer can feel satisfied even with the present improved figures. When we observe that the increase in the death-rate from the 12.1 of 1921 to the 12.8 of 1922 means that 27,600 more people died in the second year than in the first, we realise the enormous significance of a decimal point upon the public health. The time will come when we shall so much better know how to cope with influenza that the thousands who now die, as they died last year, from the epidemics will be represented by hundreds. That influenza is a preventable disease is beyond question, but we cannot expect suddenly to prevent it, and in the meantime the weak and the old fall ready victims to it. Last year, indeed, the death-rate at ages over 85 was higher than in any year since 1915. It is worth noting that while the mortality per thousand between birth and ten years of age was the lowest ever recorded, and that between ten and fifteen was unchanged, beyond that age it increased. These figures suggest the possible emergence of new problems respecting health in adolescence and early maturity.

So soon, however, as we begin to plume ourselves upon the proportionate decrease of death, the obscure laws of natality step in and produce a lower birth-rate. The provisional figures for the present year show, in the official language, "the lowest birth-rate recorded in any third quarter except during the period of the war." Since 1914 there has been a progressive decline from a fraction over 24 per thousand to a fraction over 19. A number of reasons, over and above that of natural law (whatever that law may be) can be suggested for this decline, wide-spread unemployment and the growth of prudence foremost among them. The division between the prudent and the imprudent classes in this relation is very sharply defined in the London statistics. The City of Westminster, with its picked population, chiefly of the well-to-do, had a birth-rate during the quarter of 14 per thousand ; and in the crowded, and relatively poor, Borough of Finsbury the rate was 41. Whether we deplore, or rejoice in, a falling birth-rate depends, to no inconsiderable degree, upon the point of view, but even the advocates of an indiscriminate growth of the population may be comforted by the better conservation of child life. Much of that better conservation is the fruit of pre-natal care. We are by no means merely keeping alive the fit and the unfit alike. No doubt far too many of the unfit are born and arrive at such modified bodily and mental maturity as is possible to them, to become eventually, to a greater or less degree, a burden to themselves and to the State. They constitute one of the worst problems with which the Republic has to deal, and although we may be thankful for the beginning of better things that has been made, the statesman and the man of science are likely to be actively concerned with it for another generation at least. Health, we shall do well to remember, is far from depending solely upon the triumphs of medicine. These triumphs are startling, and will yet be more startling still, but housing, education, and the growth of that self-respect which is often at present deplorably stunted, are directions in which the social reformer can help hadly less effectually than the doctor and the scientific sanitarian.

